# User Preferences and Needs for Health Data Collection Using Research Electronic Data Capture: Survey Study

**DOI:** 10.2196/49785

**Published:** 2024-06-25

**Authors:** Hiral Soni, Julia Ivanova, Hattie Wilczewski, Triton Ong, J Nalubega Ross, Alexandra Bailey, Mollie Cummins, Janelle Barrera, Brian Bunnell, Brandon Welch

**Affiliations:** 1 Doxy.me Research Doxy.me Inc Charleston, SC United States; 2 College of Nursing University of Utah Salt Lake City, UT United States; 3 Department of Psychiatry and Behavioral Neurosciences University of South Florida Tampa, FL United States; 4 Department of Public Health Sciences Medical University of South Carolina Charleston, SC United States

**Keywords:** Research Electronic Data Capture, REDCap, user experience, electronic data collection, health data, personal health information, clinical research, mobile phone

## Abstract

**Background:**

Self-administered web-based questionnaires are widely used to collect health data from patients and clinical research participants. REDCap (Research Electronic Data Capture; Vanderbilt University) is a global, secure web application for building and managing electronic data capture. Unfortunately, stakeholder needs and preferences of electronic data collection via REDCap have rarely been studied.

**Objective:**

This study aims to survey REDCap researchers and administrators to assess their experience with REDCap, especially their perspectives on the advantages, challenges, and suggestions for the enhancement of REDCap as a data collection tool.

**Methods:**

We conducted a web-based survey with representatives of REDCap member organizations in the United States. The survey captured information on respondent demographics, quality of patient-reported data collected via REDCap, patient experience of data collection with REDCap, and open-ended questions focusing on the advantages, challenges, and suggestions to enhance REDCap’s data collection experience. Descriptive and inferential analysis measures were used to analyze quantitative data. Thematic analysis was used to analyze open-ended responses focusing on the advantages, disadvantages, and enhancements in data collection experience.

**Results:**

A total of 207 respondents completed the survey. Respondents strongly agreed or agreed that the data collected via REDCap are accurate (188/207, 90.8%), reliable (182/207, 87.9%), and complete (166/207, 80.2%). More than half of respondents strongly agreed or agreed that patients find REDCap easy to use (165/207, 79.7%), could successfully complete tasks without help (151/207, 72.9%), and could do so in a timely manner (163/207, 78.7%). Thematic analysis of open-ended responses yielded 8 major themes: survey development, user experience, survey distribution, survey results, training and support, technology, security, and platform features. The user experience category included more than half of the advantage codes (307/594, 51.7% of codes); meanwhile, respondents reported higher challenges in survey development (169/516, 32.8% of codes), also suggesting the highest enhancement suggestions for the category (162/439, 36.9% of codes).

**Conclusions:**

Respondents indicated that REDCap is a valued, low-cost, secure resource for clinical research data collection. REDCap’s data collection experience was generally positive among clinical research and care staff members and patients. However, with the advancements in data collection technologies and the availability of modern, intuitive, and mobile-friendly data collection interfaces, there is a critical opportunity to enhance the REDCap experience to meet the needs of researchers and patients.

## Introduction

### Background

Accurate and complete health outcome data directly from patients or study participants (hereon referred to as *patients*) are critical for health care and research [1-3]. Unfortunately, it can be burdensome to extract patient-reported health data that researchers or providers need [4,5]. Collecting patient-reported outcomes data is becoming increasingly important in clinical research and care [6,7]. Self-administered web-based questionnaires, which patients can complete at a clinic or at home, are becoming a conventional approach to collect data for clinical research. Web-based questionnaires have advantages of being low-cost and easy to deploy at scale. A variety of clinical research electronic data capture (EDC) tools exist to streamline remote data collection and management. These systems comply with privacy regulations, integrate with different tools (such as electronic health records [EHRs]) for efficient data collection, and reduce the effort of sharing data [8]. However, user experience, cost, and maintenance of such commercial EDC systems are often prohibitive. An understanding of user experiences and preferences regarding EDC tools is critical in assessing stakeholder needs, satisfaction, and challenges in clinical and research settings.

REDCap (Research Electronic Data Capture; Vanderbilt University) is a global, secure web application for building and managing EDC for clinical research [9,10]. Developed by Vanderbilt University, REDCap is freely available for its consortium members (ie, network of nonprofit collaborators and supporters), who have an established agreement with the university. REDCap is compliant with global privacy regulations (such as the Health Insurance Portability and Accountability Act [HIPAA] of 1996) and used by more than 2.2 million researchers in more than 140 countries [9]. REDCap allows researchers to build and conduct electronic surveys, track and manage study information, schedule visits, and manage databases that are fully customizable and at no cost [11]. REDCap is designed to support data capture for research studies, providing (1) an intuitive interface for validated data capture, (2) audit trails for tracking data manipulation and export procedures, (3) automated export procedures for seamless data downloads to common statistical packages, and (4) procedures for data integration and interoperability with external sources.

Although REDCap is widely used, user needs and preferences of EDC via REDCap have rarely been studied [12,13]. For example, 1 usability study of a REDCap-based patient-facing intervention reported that patient participants found REDCap useful and easy to use but showed concerns about wordiness and inconsistent visual design [13]. Researchers have reported frequently on the implementation, use, and interventions using REDCap [10,14-20]. Understanding the preferences and needs of REDCap administrators and researchers using REDCap to capture data could help enhance existing features and EDC processes in general. While REDCap is a robust clinical research data management system, this study solely focuses on the experience of REDCap as an EDC tool. To the best of our knowledge, such preferences have not yet been studied.

### Objective

The aim of this study was to survey REDCap administrators and researchers in the United States to assess their experience with REDCap, including perspectives on advantages, challenges, and suggestions for enhancement.

## Methods

### Study Settings and Respondents

We conducted a web-based survey with representatives of member organizations listed as REDCap Partners on the REDCap website [21]. The roles of the listed members were unclear at the time of invitation sent via email. The email communication included information related to the study goals, voluntary participation, and a link to the REDCap survey. Respondents were compensated with a US $10 electronic gift card for completing the survey.

### Ethical Considerations

This study was reviewed and approved as exempt human subjects research by the Medical University of South Carolina Institutional Review Board (Pro00082875).

### Survey Design

We developed a web-based survey with multiple-choice and free-response questions (Multimedia Appendix 1) to capture the perspectives of researchers and administrators from participating REDCap consortium organizations. Our research team includes experts in biomedical informatics, behavioral sciences, mixed methods research, and user experience. The survey included 4 sections, as follows:

- *Demographics*: multiple-choice questions capturing participant role in their respective organization (Q1) and organization use of REDCap (Q2)
- *Quality of patient-reported data collected via REDCap*: Likert-scale questions capturing perspectives (ranging from 1=strongly agree to 5=strongly disagree) on the accuracy, reliability and completeness of data reported using REDCap (Q3)
- *Patient experience with REDCap*: Likert-scale question focusing on perspectives (ranging from 1=strongly agree to 5=strongly disagree) on REDCap usability, including ease of use, success rate, and completion time (Q4).
- *Data collection experience*: Free-response questions asking about the advantages (Q5), challenges (Q6), and suggestions of enhancements related to data collection, patient experience, and engagement (Q7).

### Data Collection and Analysis

We collected and managed study data using REDCap EDC tools hosted at the Medical University of South Carolina [22,23]. We generated plots and univariate statistics to summarize the data (eg, frequencies, means, SDs, and percentages). We conducted 1-way ANOVA tests to determine differences in data quality and patient experience variables by participant role and REDCap use duration. For the ANOVAs, the primary role variable was restructured to include “Educators” in the “Other” category due to the low sample size (n=1). Excel (Microsoft Corp) and SPSS (version 29; IBM Corp) were used for analyses. Free-response questions were qualitatively analyzed to identify emerging themes related to REDCap data collection experience [24]. We randomly selected 15% of the responses for initial coding and codebook development. The coding unit was done by the entirety of the participant entry. Thematic analysis of all qualitative data was done over 4 iterations using MAXQDA, during which emergent themes were identified. While the research team reviewed and honed the codes and codebook, 1 team member coded and finalized thematic coding. Discrepancies were resolved through consensus. Emergent themes were organized by frequency and topic, allowing for further qualitative analysis using complex coding query to determine concurrent themes. We reported the total frequencies per code, which may not align with the number of participants. For example, 1 participant may report a code multiple times throughout their response [25]. While thematic analysis allows us to identify principle emergent themes, it also can help identify uncommon trends that may be significant but would require further investigation in follow-up research [26]. Responses from incomplete surveys with missing quantitative or qualitative responses were excluded from the analysis

## Results

### Demographics

Between October and November 2020, 3058 representatives from 1676 REDCap member organizations in the United States were invited to complete the survey. In total, 285 (9.3%) invitees started the survey, of which 207 completed the survey. Most (150/207, 72.5%) respondents were REDCap administrators, followed by researchers (25/207, 12.1%). Furthermore, 1 (0.5%) respondent was an educator and 31 (15%) respondents served in other roles, including IT directors and managers, research coordinators and managers, program managers, project managers, director of research, library directors, and data analysts. Respondents reported that their organization had used REDCap for <5 years (92/207, 44.4%), 5 to 10 years (83/207, 40.1%), or >10 years (32/207, 15.5%).

### Quality of Patient-Reported Data Collected via REDCap

**Figure 1:**
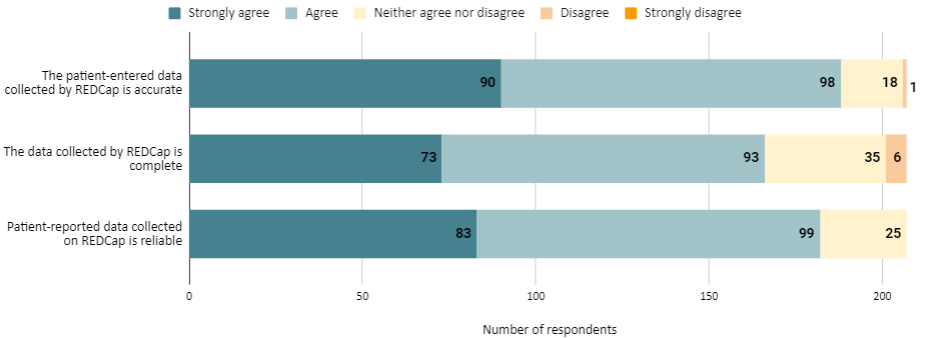
Quality of patient-reported data collected via REDCap (Research Electronic Data Capture).

We asked respondents about their perspectives of the quality of the survey data, including the accuracy, reliability, and completeness of the data collected using REDCap (Figure 1). Most respondents strongly agreed or agreed that the data collected via REDCap are accurate (188/207, 90.8%), reliable (182/207, 87.9%), and complete (166/207, 80.2%). We observed no statistically significant group differences in accuracy (*F*_2,204_=1.003; *P*=.37), completeness (*F*_2,204_=0.243; *P*=.78), or reliability (*F*_2,204_=0.245; *P*=.78) among respondent role groups. Furthermore, we observed no statistically significant group differences in accuracy (*F*_2,204_=0.672; *P*=.51), completeness (*F*_2,204_=0.045; *P*=.96), or reliability (*F*_2,204_=1.712; *P*=.18) among REDCap use groups.

### Patient Experience With REDCap

**Figure 2:**
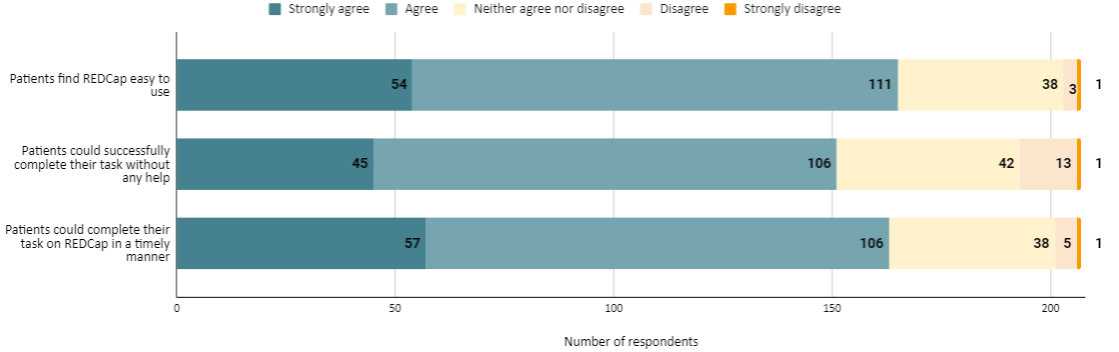
Patient experience with REDCap (Research Electronic Data Capture).

We also asked respondents about their perspectives on patient experiences with completing surveys using REDCap. Figure 2 summarizes their responses. More than half of respondents strongly agreed or agreed that patients find REDCap easy to use (165/207, 79.7%), could successfully complete tasks without help (151/207, 72.9%), and could do so in a timely manner (163/207, 78.7%). We observed no statistically significant group differences in ease (*F*_2,204_=2.025; *P*=.13), successful task completion (*F*_2,204_=0.671; *P*=.51), or timely task completion (*F*_2,204_=2.303; *P*=.10) among respondent role groups. Furthermore, we observed no statistically significant group differences in ease (*F*_2,204_=0.711; *P*=.49), successful task completion (*F*_2,204_=1.851; *P*=.16), or timely task completion (*F*_2,204_=2.000; *P*=.13) among REDCap user groups.

### REDCap Advantages, Challenges, and Enhancement Suggestions

We asked respondents about the advantages, challenges, and suggestions for future enhancements using free-response questions. The analysis yielded 8 primary codes: survey development, user experience, survey distribution, survey results, training and support, technology, security, and platform features. Within each of these themes, responses were further categorized at secondary and tertiary levels. Multimedia Appendix 2 shows the qualitative codebook with illustrative examples for each code. Table 1 shows the frequencies response classification of advantages, disadvantages, and enhancements for each code category based on respondents’ responses.

**Table 1:** Counts and percentages of response classification of REDCap (Research Electronic Data Capture) users^a^.

Code				Advantages, n (%)		Challenges, n (%)		Enhancements, n (%)
**Survey development**								
	**Design**			52 (50.5)		59 (47.6)		40 (47.1)
		Survey design	10 (9.7)		39 (31.5)		21 (24.7)	
		Response and logic	13 (12.6)		22 (17.7)		18 (21.2)	
		Survey setup	20 (19.4)		2 (1.6)		0 (0)	
		Flexibility	7 (6.8)		1 (0.8)		3 (3.5)	
		Organization	1 (1)		1 (0.8)		0 (0)	
		Testing	0 (0)		0 (0)		3 (3.5)	
	**Customizations**			7 (100)		17 (65.4)		15 (65.2)
		Language support	0 (0)		9 (34.6)		8 (34.8)	
	Project interactions			0 (0)		3 (100)		6 (100)
	Feature suggestions			1 (100)		16 (100)		49 (100)
**User experience**								
	**Usability**			105 (55.9)		29 (61.7)		8 (53.3)
		Ease of use	57 (30.3)		7 (14.9)		1 (6.7)	
		Accessibility	1 (0.5)		1 (2.1)		3 (20)	
		Intuitiveness	3 (1.6)		2 (4.3)		1 (6.7)	
		User-friendliness	11 (5.9)		4 (8.5)		2 (13.3)	
		Reliability	3 (1.6)		0 (0)		0 (0)	
		Simplicity	8 (4.3)		4 (8.5)		0 (0)	
	**User interface**			9 (52.9)		27 (50.9)		33 (52.4)
		Visual interface	2 (11.8)		5 (9.4)		29 (46)	
		Devices	5 (29.4)		9 (17)		0 (0)	
		Functionality	1 (5.9)		8 (15.1)		0 (0)	
		Design configuration	0 (0)		4 (7.5)		1 (1.6)	
	**Mobile experience**			20 (60.6)		16 (64)		21 (50)
		Ease of use	3 (9.1)		0 (0)		0 (0)	
		Interface	4 (12.1)		5 (20)		3 (7.1)	
		Mobile friendly	5 (15.2)		0 (0)		3 (7.1)	
		Mobile app	1 (3)		4 (16)		15 (35.7)	
	**Patient experien** **ce**			34 (55.7)		13 (48.1)		5 (62.5)
		Convenience	10 (16.4)		0 (0)		0 (0)	
		Engagement	9 (14.8)		6 (22.2)		3 (37.5)	
		Patient input	3 (4.9)		8 (29.6)		0 (0)	
		Patient log-in	3 (4.9)		0 (0)		0 (0)	
		Efficiency	1 (1.6)		0 (0)		0 (0)	
		Empowerment	1 (1.6)		0 (0)		0 (0)	
	Researcher experience			8 (100)		0 (0)		0 (0)
**Survey distribution and reminders**								
	**Invitations and scheduling**			20 (52.6)		25 (53.2)		25 (54.3)
		Automated scheduling and messaging	5 (13.2)		1 (2.1)		2 (4.3)	
		Save and return	3 (7.9)		15 (31.9)		9 (19.6)	
		Invitation approaches	9 (23.7)		6 (12.8)		5 (10.9)	
		Calendar integration	1 (2.6)		0 (0)		3 (6.5)	
		Patient opt out	0 (0)		0 (0)		2 (4.3)	
	**Reminders**			7 (87.5)		7 (87.5)		7 (63.6)
		Email text	0 (0)		0 (0)		1 (9.1)	
		Follow-up with patients	1 (12.5)		1 (12.5)		3 (27.3)	
	Easy distribution			11 (100)		0 (0)		3 (100)
**Results and data**								
	Results view			5 (100)		0 (0)		4 (100)
	Data sharing			8 (100)		0 (0)		3 (100)
	Data quality			3 (100)		1 (100)		0 (0)
**Training and support**								
	Education and training			7 (100)		7 (100)		8 (100)
	Support			8 (100)		15 (100)		18 (100)
	Patient support			2 (100)		12 (100)		16 (61.5)
	Patient education and communication			0 (0)		0 (0)		10 (38.5)
	Patient feedback			1 (100)		0 (0)		6 (100)
	User misunderstanding and error			3 (100)		8 (100)		1 (100)
**Technology and accessibility**								
	Consent			5 (100)		0 (0)		3 (100)
	Technology integration			11 (100)		1 (100)		12 (100)
	Technology access			17 (100)		51 (100)		0 (0)
	Technology literacy			1 (100)		33 (100)		0 (0)
**Security**								
	Privacy and compliance			15 (100)		2 (40)		2 (100)
	Trust in technology			0 (0)		3 (60)		0 (0)
**Platform features**								
	**Data collection**			14 (56)		4 (50)		5 (50)
		Comprehensive	5 (20)		0 (0)		0 (0)	
		Data administration	3 (12)		1 (12.5)		3 (30)	
		Offline access	1 (4)		3 (37.5)		2 (20)	
		Familiarity	2 (8)		0 (0)		0 (0)	
	Cost			10 (100)		0 (0)		0 (0)
	Comparison with other platforms			3 (100)		5 (100)		0 (0)
No input				7 (100)		22 (100)		52 (100)

^a^Due to the coding process (eg, double coding), the total number of secondary and tertiary codes may not add up to the primary code or 100%. The percentages are calculated based on the total number of codes in secondary and tertiary categories.

### Survey Development and Customization

Respondents perceived that REDCap surveys were generally easy (20 codes) and quick (2 codes) to set up, build, organize, and maintain (2 codes). One participant commented on these topics, “Easy to build surveys Easy to make questions easy to answer Easy to build branching questions.”

However, respondents also noted that incorrect setup by the study staff and limited default formatting options and flexibility could be challenging in developing and completing surveys (3 codes).

While some respondents pointed out that REDCap provides continuous releases with new features (2 codes) and various design and automation options to ask a variety of questions for efficient data collection (8 codes), respondents frequently pointed out the value of well-designed survey instruments in gathering high-quality information and engaging patients. They reported that complex, poorly designed surveys and ambiguous instructions (39 codes) could result in poor patient experience, potentially impacting the survey response rate and quality of data gathered. Respondents provided suggestions for enhancing survey design capabilities to streamline survey design and layout for the patients (including simplifying survey formatting, survey nesting abilities, and use of embedded fields). Respondents also suggested pilot testing of surveys before sending them out to patients (3 codes) and for study teams to follow best practices and guidelines to be more informed in survey methodologies and development. For example, 1 respondent commented:

Study teams following best practices with survey methodology and design, which can involve keeping surveys short & sweet, choosing appropriate field types for the question at hand, phrasing questions and response options well to reduce mental burden and make it easier for patients to answer questions.

Respondents also reported that the availability of various response types, data validation, and branching logic ensure high-quality data collection (13 codes). One respondent commented on this advantage, “The wide array of validations can help patients enter data correctly.”

Another respondent noted similarly, “Data validation and branching logic make participants conform to data standards and allows researchers to obtain higher quality data.”

While data validation was discussed positively, respondents more frequently noted the challenges with response and logic types (22 codes), often pointing out that the actual response and logic types available from REDCap are not conducive to good survey design. One respondent made a clear reference to this issue saying, “It all depends on who sets up the survey, but until recently it has been a challenge to create grids of disparate data entry fields.”

In addition, some respondents noted that due to the logic types, patients can make critical mistakes affecting the completeness of the data:

...branching logic at a very question to determine if they qualify or not. Sometimes, they accidently select different value in a hurry, and the survey gets completed. It is hard for them to change the response or refill the survey without admin help.

Respondents noted many enhancement potentials within this category, such as voice input (4 codes), superior data entry experience (5 codes), use of a more conversational approach in response types (1 code), more effective multimedia (5 codes), and gamification of survey (2 codes). While REDCap offers multimedia options, respondents often suggested that options become more interactive and effective:

...more visual aids in questions, and the ability to answer with images. For example, by painting the areas afflicted on an image.

One respondent explained how multimedia may be further useful:

...ability to add images to response options. Especially when working with minorities (traffic lights, or smiley faces).

In addition to the design of the surveys, respondents noted that while REDCap surveys are readily customizable (7 codes), there are far more reported challenges (17 codes) and need for enhancements (15 codes). Respondents noted customization was not possible in some cases: “Default formatting options are limited.”

However, many respondents focused on the lack of multi-language support (9 codes) as the critical challenge:

...multi-linguistic support. This is always a challenge for any software system/platform, and REDCap is no different...

They frequently suggested enhancements to include multi-language support (8 codes) and customizations in forms’ appearance (6 codes). For example, 1 respondent mentioned, “Allow for some more customization of the overall look/feel of surveys.”

With respect to challenges with survey interactions, respondents reported that REDCap capabilities at the time did not send new surveys or allow patients to complete future surveys if previous surveys were incomplete (2 codes). One respondent mentioned the following:

...[t]he longitudinal design functionality in REDCap requires a participant to take each form before moving to the next, but our experiment design does not require this, and sometimes people will miss sessions and need to move on to the form for the next one. But if we stack all of the forms in one event, we cannot direct people to an individual form, only to the queue.

One participant commented on REDCap’s *“*inability to provide staff log-in status*.”* (1 code). Respondents requested features for internal messaging or chat between study staff (2 codes), enhancing flow and cross-linking between projects (2 codes), ability to easily add study staff members outside of the organization (1 code), and ability for patients to skip longitudinal surveys (1 code).

### User Experience

Respondents perceived REDCap to be easy to use for both patients (ie, to take surveys) and the study staff (ie, to build and distribute surveys; 57 codes). One respondent commented as follows:

REDCap is the easiest way to survey patients, families, and staff who are not part of our study team. We would not be able to conduct these surveys without it!

They also perceived REDCap to be user-friendly (11 codes), simple (8 codes), intuitive (3 codes), timely (2 codes), and reliable (3 codes). Although some respondents reported REDCap allows for quick data collection (7 codes), they perceived that lengthy or poorly designed surveys (eg, too many clicks and not enough instructions) could lead to fatigue and poor participation (15 codes). While the usability perceptions were generally positive, respondents reported that the platform was not as user-friendly or outdated as other commercial data collection platforms (4 codes), unintuitive (2 codes), and clunky for study staff (4 codes). They reported that *“*REDCap is not the simplest tool to learn how to use*”* for study staff (4 codes) and patients (3 codes). Respondents suggested the need to enhance accessibility features, such as the ability to change font size, screen reader view, and text-to-voice, among others (3 codes). In total, 8 (3.9%) of 207 respondents reported that the REDCap interface was advantageous for study staff considering its consistent interface and automated features, which reduce burden.

Respondents generally reported REDCap’s visual user interface as challenging to use. Although some respondents perceived the interface to be clean or simple looking (9 codes) and optimized for various devices (5 codes), other respondents perceived that REDCap’s interface was not modern looking (7 codes) or appealing (5 codes). One respondent mentioned, “The web interface of our survey pages are very basic, and narrow,” whereas another respondent said, “[REDCap has] Very set layout of each item, can’t make it look more ‘modern’ like other websites are at this time.”

Respondents considered REDCap as not having a configurable design (4 codes) and some noted the user interface’s poor functionality (8 codes). One respondent described both issues when explaining the challenges of the user interface:

REDCap is simply not user friendly in any way. The data structures are often too rigid and frankly outdated in being an effective tool for data collection.

Respondents suggested the redesign of the REDCap user interface to be consistent with modern data collection platforms (27 codes), options to change the visual appearance and formatting of the surveys (3 codes), adding progress tracking aids (such as an automatic progress bar) for patients (2 codes), and a more flexible interface (1 code).

Some respondents appreciated REDCap’s mobile access (4 codes), availability of mobile apps for study staff (REDCap mobile app; 2 codes) and patients (MyCap; 6 codes) supporting offline data collection, and perceived REDCap to be easy to use on mobile devices (3 codes) and mobile friendly (5 codes). While respondents appreciated the mobile interface, they reported that the mobile experience is affected by poor and suboptimal mobile user interface and scaling on smaller screens (5 codes). One participant reported the following:

We design our surveys on a computer, but many of our participants use their phones. We try to check how answers scale when the screen size changes, but some phones rescale to a different aspect ratio leading to challenges.

They also reported that although the REDCap mobile app is available for study staff, it is not ideal and is difficult for study staff to set up the app (4 codes). One respondent mentioned the following:

I think that the REDCap mobile app is a bit too far separated from the web version, in as much as there is no access to external modules and other important features.

Respondents suggested a need for an enhanced mobile app and interface (21 codes), including advanced capabilities for the study staff to view study records and perform analysis (2 codes) and push notifications (2 codes). One respondent mentioned the following:

[They need] better workflows with mobile phones, like notifications instead of just text messages. Something like an App except not the current one which is focus on asymmetric internet access.

Respondents also commented on patient experience with REDCap. Overall, respondents noted that REDCap makes it easier for patients to complete the surveys at their convenience (10 codes), all while increasing engagement levels (9 codes). They saw REDCap as a way to make data collection more efficient and empowered (2 codes), especially as patients did not need to register or remember usernames or passwords to use the platform (3 codes). One participant said, “[Survey] Can be done at the patient’s convenience from any digital device.” A common challenge reported was the patient’s desire and motivation to complete the surveys, being able to use the platform, and fatigue with lengthy surveys (13 codes). Suggestions for improving patient experience included maintaining engagement using visual aids and gamification (3 codes), a patient dashboard to keep them up to date on status of longitudinal studies (1 code) and making the platform more patient friendly (1 code). One respondent commented as follows:

For longer surveys, having a way of maintaining engagement by making the surveys more interactive (e.g. fun feedback to participants as they progress) would be nice. Some periodic messages of encouragement like “Great job!” “Keep it up!”

### Survey Distribution and Reminders

Respondents found it advantageous that REDCap included multiple ways to invite patients, such as emails or embedded links (4 codes). REDCap surveys were easy to distribute (11 codes) and could be automated and scheduled on a timeline easily. One participant commented on this aspect, “It can send surveys to participants directly, and on a schedule when the project is longitudinal.” REDCap’s ability to send patients custom links was an advantage respondents liked (3 codes): “For online surveys: able [to] send individualized email links...automated email with message that has piping upon completion.” One respondent pointed out that there was *“*no scheduling component for visits*”* and suggested this feature. One respondent suggested the ability to send attachments with automatic notifications.

In addition, the ability to send completion reminder emails to patients was reported to reduce the burden on clinic staff while engaging patients (7 codes). Reminders also allowed the study staff members to follow up on incomplete surveys but 1 respondent mentioned that this was challenging while respondents suggested for improvements in customizing reminders and enhanced tracking for incomplete surveys longitudinally (3 codes):

If there was a more efficient way to upload and manage patient invitations, as well as identify which patients have completed the survey within previous xx months therefore a new survey invitation does not need to be sent.

Respondents noted patients sometimes missed invitations and reminders because email service providers blocked the emails (2 codes): “We have had email providers block REDCap emails, specifically Yahoo.com email.” There was also confusion about the email sender as the emails were “from” REDCap instead of the study staff (2 codes):

From my experience... The emails that are sent out to respondents are not user friendly. The ‘From’ text box comes from REDCap, not from my email address.

In addition, this respondent noted the emails were not user-friendly, sometimes arriving with broken links going to patient’s junk mail, and requiring patients create a completely new log-in to complete a survey. One respondent suggested REDCap may “make it easier to send mass emails that are individually linked with the patient’s profile; create a prettier or more visually appealing interface for patients.” Furthermore, integrations to link communications to personal calendars were thought to be beneficial (3 codes). Respondents wanted a way to automatically opt out patients from surveys that were being distributed over a period (2 codes). One participant stated they, “would really like to be able to set a flag for opt-out subject [s] when distributing surveys over a period of time. We currently have to remove their emails to prevent future distribution.”

Respondents commented on the “Save and Return” feature (25 codes), which allows patients to leave and return using a unique code to complete the survey at a later time. Although REDCap’s Save and Return feature existed, respondents noted that this feature was often difficult to use (15 codes). They reported that patients may forget or not save their return code or may not know how to return to the survey, resulting in incomplete data or delay in data collection. One respondent commented, “It is not always obvious how to ‘save and return later’ if that is an option or even be aware that that is an option.” Respondents suggested improvements (9 codes) to send the unique save and return code via emails, with reminders and save in invitation logs such that the study staff could provide it to patients if needed. In addition, respondents suggested that improving user-friendliness and patient awareness of this feature could increase response rates and data completion. A participant noted the following:

If they [patients] don’t complete the survey the first time they often forget their return code and lose it. It would really help if the reminder emails had the return code, of if it could be included on the survery [sic] invitation log page that would make it much easier to find and give to the patient.

### Results and Data

Respondents liked that REDCap made data exportation easy for storage and analysis purposes (8 codes). Not only was it easy to export data out of the REDCap survey tool, it also made the analysis of the data much easier for the study staff, even those with minimal statistics training. As 1 respondent put it, “[REDCap has a] good translation into a dataset [and] easy statistics for those with minimal statistical training.” Respondents (4 codes) pointed out the need for improving data exports and seamless communication with third-party solutions to send and receive information:

Being able to send to communicate and receive information from other software programs like Clinical Conductor for Demographic information and seamless data uploads.

Respondents perceived that it is easy to create reports and monitor patient responses on REDCap and review specific data points (5 codes). Some respondents provided suggestions to edit charts and graphics as well as being able to share user- or survey-specific data (3 codes). For example, 1 respondent mentioned the following:

Ability for researchers to edit/modify graphics that can be automatically displayed with reports within redcap. This would facilitate researchers’ ability to use those charts.”

Another respondent mentioned, “built in tools to share summary-level data (you vs the whole study) or findings.” While respondents perceived that REDCap allows capturing accurate and complete high-quality data (3 codes), 1 respondent mentioned the following:

As with every self-service data entry portal accuracy of self-service data entry is wildly unreliable. There is real value to having a trained rep assisting the client enter information, when possible.

### Training and Support

Respondents reported that REDCap’s active online community and support allowed REDCap users (including administrators and researchers) to find information and answers on how to manage, design, and conduct surveys (8 codes): “...it has a huge user base and a great consortium full of all the information you need to begin administering [surveys].” Respondents mentioned needing REDCap or IT support for patients to complete consent forms or surveys (12 codes). Although support existed for survey designers and administrators, it did not extend to patients completing surveys. Respondents suggested REDCap needed a way to educate or support patients in completing surveys (10 codes) and obtain help via on-demand messaging to study staff members (2 codes). As 1 participant suggested, REDCap should allow patients to “Click icon and get video explaining any information on a field*.*” Another participant asked that REDCap have the following:

Dedicated instrument defined support button at the top that takes participants to a page made by the study team where we can put in a zoom room link monitored by study staff, phone numbers, or some pointers on definitions/examples on the instrument.

Respondents also suggested a need for obtaining standardized patient feedback surveys to better engage them and understand their experience (6 codes).

Although some respondents mentioned REDCap required minimal training to get started (7 codes), some respondents (especially REDCap administrators) mentioned the need for training survey designers to set up REDCap tools and surveys to design high-quality surveys (7 codes). When asked about challenges, 1 participant mentioned the following:

Lack of resources for support (in person- phone) and functionality. It is not always easy and takes a lot of time to build tools. Not able to use to its fullest capacity or correctly—basically training ourselves. Library or community network does not help either. Not knowing how to set up properly more complicating functions inhibits usage.

Respondents suggested more information and mandatory training for survey builders, including better guidelines and training videos to enhance builder and patient experience (8 codes). Respondents also perceived that patients taking surveys often do not understand how to fill out surveys or certain questions (8 codes) and having expert survey designers and well-designed surveys could alleviate these concerns (1 code).

### Technology

Respondents often noted challenges of access to the internet and devices (51 codes) as well as technology literacy (33 codes):

Patients [without] a computer, device, or smart phone may not be able to use REDCap.

As REDCap is web based, data collection could be difficult in rural and low-resource areas due to lack of access to technology (4 codes), such as a computer or reliable internet connection. Another participant noted, “I do work in global health, so our colleagues in resource-limited settings have challenges with the internet connection.”

They also noted REDCap’s ability to integrate with other technologies, such as messaging tools (eg, Twilio) as well as open application programmable interface to be beneficial (11 codes). In comparison, 1 participant noted as a challenge that, “integrating the ReCap [sic] extract with Epic [EHR] data. But once the system is setup it’s easy to maintain.” Respondents suggested integrations with other clinical trial management systems for seamless data transfers and EHRs to conduct surveys or autopopulate patient medical information:

The only other thing that would be super cool is if it could blow surveys into EPIC for documentation when needed.

Respondents also referred to the informed consent capabilities of REDCap (8 codes). Even though they noted the consent module to be advantageous to obtain remote informed consents especially after the COVID-19 pandemic (5 codes), respondents suggested more enhancements, such as a 1-step consent process (3 codes).

### Security

Respondents commented positively on the security and compliance of REDCap (15 codes). They reported that HIPAA compliance and the ability to store patient data securely are important advantages of REDCap. One participant commented that “all client data can be stored in one HIPAA compliant platform.”

Respondents mentioned mistrust of technology (3 codes) could make patients feel uncomfortable sharing medical information on web-based platforms. One respondent commented that surveys requiring password protection are difficult for patients. They also provided enhancement suggestions (2 codes) related to maintaining HIPAA compliance, enhancing security, and assuring patients that their health information is safe and secure with REDCap.

### Platform Features

Respondents found REDCap advantageous in enabling researchers to collect and patients to provide health data remotely (23 codes):“It has made it much easier for patients to submit their questionnaires and information using an online platform,” especially during and after the COVID-19 pandemic.

Respondents perceived REDCap as a comprehensive or versatile (5 codes) data collection solution noting the following: “It provides us a comprehensive tool for collecting, tracking, and managing patient data and outreach.” They also noted administration and maintenance (3 codes) to be advantageous as REDCap allows *“*being able to maintain administrative research tasks together with the data collection*.”* They noted REDCap’s offline data collection (using REDCap mobile apps) to be challenging (3 codes) and suggested that the offline feature should be improved for better data collection experience (2 codes). In addition, respondents noted the familiarity with REDCap among researchers (2 codes) and seamlessness (1 code) for the study personnel to be advantageous.

In addition, REDCap being available for free to REDCap consortium members was sought to be beneficial (10 codes). While some respondents noted REDCap being simpler and easier than other commercial platforms and paper forms (3 codes), some also noted that REDCap’s interface was not easy to use or user-friendly compared to modern data collection tools (5 codes).

### No Input

Respondents did not provide inputs with respect to advantages, disadvantages, and enhancement suggestions stating lack of experience or ability to provide inputs or not using REDCap for patient data collection (81 codes). Some nonsensical or unrelated comments lacking information context or irrelevant responses were excluded from the analysis. For example, when asked about enhancement suggestions for REDCap, 1 participant responded, “To REDCap or??.”

## Discussion

### Overview

This study aimed to identify the advantages, challenges, and future opportunities for enhancements from the perspectives of REDCap administrators and researchers. To the best of our knowledge, this is one of the early studies of user perspectives on REDCap services and features. We believe that the findings of this study will aid REDCap developers and consortium users in better understanding stakeholder needs to enhance and customize REDCap features as well as researchers in improved survey development and data collection.

### Principal Findings

Respondents had overwhelmingly positive perceptions of REDCap’s survey design and data collection interface. The vast majority of respondents agreed or strongly agreed that data collected via REDCap were accurate (188/207, 90.8%), reliable (182/207, 87.9%), and complete (166/207, 80.2%). They found REDCap advantageous as it is free for its consortium members, secure, and easy to use. Respondents also perceived REDCap as easy and flexible to create and customize surveys including a variety of response and validation options, which make data collection easier for survey takers. However, respondents pointed out that poor survey design—often attributed to human factors (eg, lengthy forms and lack of knowledge among study staff) or technology limitations (eg, restrictions in survey and visual formatting in REDCap)—could result in poor patient experience and, ultimately, response and completion rates. Optimal design of survey forms is critical for assuring patient comprehension of the forms and accurate data collection [27,28]. Furthermore, direct investigations of REDCap user experiences and preferences could allow better understanding of the need for study staff and patient education. In addition, further research related to user needs for survey development and optimization can lead to enhancing their experience of developing high-quality surveys. One respondent pointed out the following:

It [REDCap] needs a much better understanding of how users engage the questions on a form (e.g., sit and watch users and staff try to figure out acceptable data type entries!). Needs a solid revamping in how it works “out front” and to run a series of user groups—patient and staff.

Although respondents appreciated the availability of REDCap’s community support for administrators and study staff, they pointed out that REDCap has room for improvement in this realm: the tool is not simple to learn, and there is a need for more training of study staff to help develop efficient, unambiguous survey instruments that can enhance patient experience. Poorly designed surveys and questions could potentially lead to incomplete responses and inaccurate data. Respondents pointed out the need for supporting patients, especially to ensure they understand the questions and can obtain help when needed in filling out surveys. Direct help from study staff members to fill out surveys or having the ability to directly send a message to study staff could alleviate misunderstandings and errors in completing surveys. Previous research suggests that the ability to obtain clarifications about survey questions can enhance response accuracy [28]. Further research and availability of resources are necessary to guide study staff members in creating well-designed instruments. In addition, understanding the factors affecting patients’ experience in completing REDCap surveys and reasons for misunderstanding and errors could also enhance the REDCap experience and health data collection processes.

Opinions on patient experience and usability were more mixed. Most respondents agreed or strongly agreed that patients found REDCap easy to use (90.4%), able to be completed without assistance (79.8%), and able to be completed in a timely manner (87.5%). These strongly positive perceptions of REDCap usability are consistent with a prior study in which 6 out of 7 participants needed no help using REDCap, achieved 71% to 100% task completion, and provided 89% positive reaction words [13]. Qualitative outcomes showed that respondents perceived REDCap made it convenient for patients to provide data remotely without having to log in or remember credentials. Although they commented that patients can complete REDCap surveys using a device of choice (such as a laptop or mobile), technology access and technology literacy appeared to be a concern. Living in rural or low-income areas also presented issues for survey access. Respondents noted low-resource areas without stable internet access meant data collection was not reliable. Lack of internet access not only meant surveys could not be accessed but also meant the data collection process could be interrupted. REDCap’s MyCap and REDCap Mobile app can allow study staff and patients to complete the collection of data offline, but they were also deemed challenging due to the lack of features compared with the web interface. In a study by Doyle et al [19], the REDCap mobile interface was less favorably received by participants. Similarly, REDCap’s *Save and Return* feature allows users to complete surveys at a later time, which could be helpful during poor internet access; however, respondents recommended enhancements in the feature to improve patient experience, specifically an easier way for patients to remember and retrieve the return code. One participant noted this difficulty that patients face in attempting to use the feature:

If they don’t complete the survey the first time, they often forget their return code and lose it. It would really help if the reminder emails had the return code, or if it could be included on the survey invitation [log-in] page...

It is imperative to better understand patient and research participant experience with REDCap in completing surveys via larger and direct studies.

This study identified opportunities to improve the usability of REDCap. Respondents suggested enhancements in the patient-facing survey user interface to be in line with present EDC tools on the market, wanting a sleeker, modern, and cleaner looking interface. A variety of EDC tools are available for health care and non–health care data collection providing modern, device-friendly, and intuitive user interfaces to promote patient engagement [29-32]. In recent years, virtual conversational agents or chatbots have emerged as intuitive and engaging mediums for data collection. Modern data collection tools allow survey designers to develop chatbot-based interactions to collect health data mimicking human-to-human conversations. Studies have shown that individuals prefer chatbot-based conversational data collection experience in comparison to traditional web-based forms [33,34]. Visual and graphical enhancements in REDCap appearance of surveys, patient communication, and researcher interface could support modernization of REDCap-based surveys, thus providing study staff and patients with clear and effective experience of health data collection.

Respondents wanted the mobile interface updated to look more like other commercial products, such as Qualtrics or SurveyMonkey. As more individuals are using mobile devices to obtain health information, it is of great importance to enhance their experience with mobile data collection [35]. They also suggested that the mobile apps have similar features as the web-based REDCap. Other requests included REDCap to support more languages or a translation service, where surveys could be translated to patients’ preferred languages. Though it has some language capabilities, including Spanish, respondents wanted more language options built into REDCap. In addition, there was concern about the literacy of patients leading to suggestions for REDCap to include tools allowing patients with various literacy levels to access surveys. Respondents suggested inclusion of voice capabilities and more multimedia and gamification features in response options, such as a picture interface where patients could locate their pain visually for researchers. Inclusion of these features could further enhance the experience among patients with higher accessibility needs and low literacy. We also noted that some respondents suggested features that were available within REDCap at the time of conducting the survey. Suggestions included availability of REDCap’s mobile version, embedded fields for responses, and integrations with messaging services such as Twilio. This again points out the need for education among study staff and organizational administrators to enable the optimal and effective use of REDCap features.

### Limitations

This study is not without limitations. Although we recruited over 200 respondents, the sample size is small in comparison with the existing user base. We recruited fewer researchers (25/207, 12.1%) than administrators (150/207, 72.5%) who may be more directly involved in survey design and data collection. We also did not ask for participants’ training and experience with REDCap. Future studies should focus on better understanding user perspectives (especially researchers) while also considering the type and amount of REDCap training received by the user. We asked individuals’ opinions that are valuable but may be subject to bias, incomplete recall, or lack of information. For example, we asked information about their institution’s REDCap use, but we did not include a response option or decline responding if they did not have accurate information. We also used REDCap as the platform to conduct our survey, which may have potentially biased responses by familiarizing participants with REDCap more than necessary. Participants’ free-ended responses may have been influenced by how our study was designed or how the features were used. Future, more direct studies are warranted to better understand preferences. We recruited respondents from current REDCap consortium members, who may be more likely to believe REDCap is highly usable, as they may act as REDCap champions within institutions. We may be missing critical information by not capturing the perspectives of people who are not frequent users or consortium members. Future research should capture opinions of novice or past REDCap users. We also did not ask for information about participants’ institutions, REDCap versions and plug-ins used, or institutional policies and customizations. It is possible that participant feedback may be related to institutional requirements or policies. Furthermore, we asked researcher and administrator opinions on patient experience. However, we did not directly assess the patient or research participant experience. Understanding patient experience is important to study in future research. In addition, a comparison of the REDCap experience with other EDC platforms could provide a better understanding of study staff and patient needs. A recent study compared individuals’ experience in completing health forms using REDCap versus a chatbot platform. The results revealed that over 69% of participants preferred a chatbot for data collection with higher usability and net promotor scores for the chatbot [33]. The chatbot provided superior engagement and interactivity and was perceived as more intuitive than a standard, web-based REDCap interface. Future studies should look into better understanding study staff and patient needs to optimize survey development and data collection experience.

### Conclusions

This pilot study aimed to assess stakeholder perspectives on experience with REDCap as an electronic health data collection tool. The findings revealed researchers and administrators perceive REDCap as a valued, low-cost resource that enables them to remotely collect and report health data in a secure and easy way. They also indicated a generally positive health data collection experience by clinical research and care staff members and patients. Although, with the advancements in data collection technologies and availability of interactive and intuitive user interfaces, there is a critical opportunity to enhance the REDCap experience to meet the needs of its vast user base of researchers and patients.

## Appendix

Multimedia Appendix 1 Survey.

Multimedia Appendix 2 Qualitative codebook.

## Abbreviations

EDC: electronic data capture
EHR: electronic health record
HIPAA: Health Insurance Portability and Accountability Act
REDCap: Research Electronic Data Capture
