# Supplementary material for: User Preferences and Needs for Health Data Collection Using Research Electronic Data Capture: Survey Study
Source: JMIR Med Inform. 2024 Jun 25;12:e49785. doi: 10.2196/49785 (PMC11234068; doi:10.2196/49785)
Supplement: Multimedia Appendix 1 [file medinform_v12i1e49785_app1.docx]

## **Appendix A. Survey**

**Demographics**

1. What is your position at your organization?
2. Research/REDCap Administrator
3. Researcher
4. Educator
5. Patient Engagement Specialist
6. Other: _____________
7. How long has your organization used REDCap?
   1. <5 Years
   2. 6-10 Years
   3. >10 Years

**Quality of patient reported data collected via REDCap**

1. Based on your experience, how would you rate the data reported by patients (or research participants) using REDCap with respect to the following?

|  | Strongly agree | Agree | Neither Agree Nor Disagree | Disagree | Strongly disagree |
| --- | --- | --- | --- | --- | --- |
| The patient-entered data collected by REDCap is **accurate** |  |  |  |  |  |
| The data collected by REDCap is **complete** |  |  |  |  |  |
| Patient reported data collected on REDCap is **reliable** |  |  |  |  |  |

**Patient experience with REDCap**

1. How would you rate the following with respect to patient (or research participant) perceptions towards REDCap?

|  | Strongly Agree | Agree | Neither Agree Nor Disagree | Disagree | Strongly Disagree |
| --- | --- | --- | --- | --- | --- |
| Patients find REDCap **easy to use** |  |  |  |  |  |
| Patients could successfully complete their tasks **without any help** |  |  |  |  |  |
| Patients could complete their tasks on REDCap in a **timely manner** |  |  |  |  |  |

1. What do you think are the biggest **advantages of REDCap** supporting patient engagement?

[open-ended paragraph box]

1. What are the biggest **disadvantages of REDCap** impacting patient engagement?

[open-ended paragraph box]

1. What **enhancements or improvements** do you believe could **improve patient engagement** with REDCap?

[open-ended paragraph box]
