# Supplementary material for: User Preferences and Needs for Health Data Collection Using Research Electronic Data Capture: Survey Study
Source: JMIR Med Inform. 2024 Jun 25;12:e49785. doi: 10.2196/49785 (PMC11234068; doi:10.2196/49785)
Supplement: Multimedia Appendix 2 [file medinform_v12i1e49785_app2.docx]

## **Appendix B. Qualitative codebook**

| **Secondary Code** | **Tertiary Code** | **Definition** | **Illustrative examples** |
| --- | --- | --- | --- |
| ***Primary Code: Survey Development*** | | | |
| Design | Survey Design | Reference to the process in which they use the tool to build assessments (i.e. layout, creation of survey tool) | *“Study teams following best practices with survey methodology and design, which can involve keeping surveys short & sweet, choosing appropriate field types for the question at hand, phrasing questions and response options well to reduce mental burden and make it easier for patients to answer questions ”* |
|  | Response and Logic Options | Various response types and branching logic capabilities to customize survey questions based on previous responses | *“REDCap's use of data quality conditionals and defined survey field types makes patient entry more reliable than paper surveys.”* |
|  | Survey Setup | The ease of creating or modifying surveys in REDCap | *“Easy to build surveys Easy to make questions easy to answer Easy to build branching questions”* |
|  | Flexibility | Flexibility of REDCap in survey development | *“[REDCap] is very flexible in terms of data capture, survey features, etc.”* |
|  | Organization | Ability to organize or maintain surveys or databases | *“...the longitudinal design functionality in REDCap requires a participant to take each form before moving to the next, but our experiment design does not require this, and sometimes people will miss sessions and need to move on to the form for the next one. But if we stack all of the forms in one event, we cannot direct people to an individual form, only to the queue.”* |
| Customization | Survey Customization | Option to create assessments that are specific to their projects, organizations, or goals | *“highly customized and tailored to almost any research project/study need”* |
|  | Language Support | Request for REDCap to support multiple languages for survey completion | *“Make REDCap easier to collect multiple languages for the same questionnaire.”* |
| Feature Suggestions | | Request or suggestion for new features | *“Audio and/or video capture of subject responses.”* |
| Project Interactions | | The ability of REDCap projects to communicate with each other | *“Flow between instruments, ability for projects to "talk with each other" to synchronize data between multiple projects,”* |
| ***Primary Code: User Experience*** | | | |
| Usability | Ease of use | Facilitators and barriers to correct use by patient and/or researcher | *“The forms are easy to create, reports are easy to create. Even the CRC can do these things. You don't need technical staff involved.”* |
|  | Accessibility | Reference to making surveys more sensible, meaningful, and usable | *“Enhanced templates with built-in accessibility options (e.g. reader-view, text-to-voice, touch-screen support)”* |
|  | Intuitiveness | Reference to whether REDCap is natural to learn or use | *“participants find online surveys fairly intuitive”* |
|  | User-friendliness | System’s capability to be easy to use and understand | *“the system is pretty user friendly”* |
|  | Reliability | The ability of REDCap to be trustworthy and perform consistently | *“survey field types makes patient entry more reliable than paper surveys”* |
|  | Simplicity | Discussion whether REDCap is simple to use or simplicity of user interface | *“It's clunky--made for researchers, not for patients.”* |
| User Interface | Visual interface | Patients and/or researchers’ experience of the layout, content, and general engagement with the interface | *“More control of survey and survey invitation aesthetics and organization. Again, being out of date is probably the biggest driver of these problems.”* |
|  | Devices | Ability to take surveys on multiple devices | *“REDCap offers the flexibility of using different devices for data collection.”* |
|  | Functionality | Ability for the interface to work allowing users to complete tasks | *“I think that the REDCap mobile app is a bit too far separated from the web version, inasmuch as there is no access to external modules and other important features.”* |
|  | Design configuration | Design features allowing for customization of interface, such as organization of data displayed | *“Sometimes the questions aren't relevant to them, either because there's a ceiling effect to the survey or because the computer adaptive question pulled from the question back doesn't easily apply to them.”* |
| Mobile Experience | Ease of use | How research patients and/or researchers experience the layout, content, and general engagement with the REDCap interface when using a mobile device and REDCap apps | *“The ReCap mobile app is also easy to deploy and push new forms to patient's phones. This enables faster survey data collection with a lower barrier to entry.”* |
|  | Interface | Patients and/or researchers’ experience of the layout, content, and general engagement with the REDCap’s mobile interface | *“We design our surveys on a computer, but many of our participants use their phones. We try to check how answers scale when the screen size changes, but some phones rescale to a different aspect ratio leading to challenges.”* |
|  | Mobile friendly | System’s capability to be easy to use and understand on mobile devices | *“Develop REDcap mobile friendly version”* |
|  | Mobile apps | Reference to mobile app versions of REDCap | *“I think that the REDCap mobile app is a bit too far separated from the web version, inasmuch as there is no access to external modules and other important features.”* |
| Patient Experience | Convenience | Ability of patients to provide data virtually at their convenience | *“Convenience for participants to complete virtually with the save and return option.”* |
|  | Engagement | Ways that patients engage with REDcap. Normally referencing pain-points or successful engagement techniques | *“track patient response enable researchers to monitor and encourage participants to engage.”* |
|  | Patient Input | Patients’ ability to report data and related experience | *“[give] participants the opportunity to provide more information than they might when speaking with a researcher.”* |
|  | Patient Login | Reference to need for patients to register or login for survey completion | *“No usernames or passwords are required.”* |
|  | Efficiency | Ability of patients to complete the surveys efficiently using REDCap | *“simple, fast, efficient for the patient and the study team”* |
|  | Empowerment | Patient empowerment in digital data collection | *“Patient empowerment”* |
| Researcher Experience | | Researcher experience with REDCap | *“Makes data collection a seamless process for all researcher and study personnel involved.”* |
| ***Primary Code: Survey Distribution*** | | | |
| Invitations | Survey Invitations | Ability to set up a project, send invitations, and schedule future surveys. | *“able send individualized email links and stop to return later;”* |
|  | Automated Scheduling and Messaging | Ability to automatically send out invitations and messages to patients. | *“REDCap allows for wonderfully robust communication with users, to meet their preferences, including automated email/text invitations and alerts & notifications.”* |
|  | Save and Return | Ability to leave and return to an incomplete survey using a unique code. | *“If they don't complete the survey the first time, they often forget their return code and lose it. It would really help if the reminder e-mails had the return code, or if it could be included on the survey invitation [*log-in*] page...*” |
|  | Invitation Approaches | Ways patients can be invited to complete surveys. | *“multiple ways of reaching out to the participant”* |
|  | Calendar Integration | Ability to integrate personal calendars. | *“Introducing or making some sort of calendar enhancement available to participants could catalyze patient engagement.”* |
|  | Patient Opt Out | Ability of patients to opt out of longitudinal surveys if not applicable. | *“unable to automatically opt out of receiving emails- if they had this option they might not get so frustrated”* |
| Reminders | Survey Reminders | Ability to send reminders to patients to complete assessments. | *“The option to remind participants regularly on an auto-scheduled basis has both reduced our workload, as well as increased patient engagement.”* |
|  | Email text | Customizations in email text for reminders. | *“Allowing for the survey reminder emails to have a different text than the original survey invite.”* |
|  | Follow Up with Patients | Ability to connect with patients to remind them about completing surveys. | *“If there was a more efficient way to upload and manage patient invitations, as well as identify which patients have completed the survey within previous xx months therefore a new survey invitation does not need to be sent.”* |
| Ease of Distribution | | Ease of sending out surveys | *“ease of distribution via email and text.”* |
| ***Primary Code: Results*** | | | |
| Results View | | How users wish to view and receive the results of their assessments. | *“reports are very helpful to look at specific data”* |
| Data Sharing and Exports | | The ability to export results and share the collected data with others. | *“Improvements or additional support with importing and exporting, and report building.”* |
| Data Quality | | Perceived reliability and accuracy of the data collected by the instrument. | *“Data validation and branching logic make participants conform to data standards and allows researchers to obtain higher quality data.”* |
| ***Primary Code: Training and Support*** | | | |
| Staff Training | Education and Training | Request for more resources or training for study staff. | *“Better training videos for teaching researchers how to build surveys. Usually poorly built surveys make things challenging for patients to take them”* |
| Support | Staff Support | Availability of support for researchers to provide clarification. | *“It has a huge user base and a great consortium full of all the information you need to begin administering REDCap.’* |
|  | Patient Support | Availability of support for patients to provide clarification. | *“Older, tech challenged or very ill patients may have trouble completing a survey alone and prefer to have the questions read and the interviewer complete the survey”* |
|  | Education and Communication | Request for more resources and ways to communicate with patients. | *“Instructional videos aimed at the participant rather than the form administrator.”* |
| Patient Feedback | | Ability to obtain patient feedback during or after surveys. | *“patient feedback is important to obtaining good quality data and continue patient engagement.”* |
| User Understanding and Errors | | Human error associated with designing an assessment as a researcher or taking an assessment as a patient. | *“Patients (and hospital staffs) often don't quite understand how to fill in surveys and forms.”* |
| ***Primary Code: Technology*** | | | |
| Consent | | Improving the informed consent procedure currently in place. | *“The eConsent Module can provide virtual consent (for both Part 11 trails and other types of research, if certified) which is especially important during COVID”* |
| Technology Integration | | Ability to integrate with specific health IT software (e.g. EHRs). | *“the only other thing that would be super cool is if it could blow surveys into EPIC for documentation when needed”* |
| Technology Access | | Access or barriers to technology patients may face. | *“easily accessible the majority of the time”* |
| Technology Literacy | | Reference to patient’s ability to use technology or complete surveys online. | *“One of the biggest challenges for patients can be using the QR code if they are not as tech-savvy or not receiving the email for survey links.”* |
| ***Primary Code: Security*** | | | |
| Privacy/Compliance | | Reference to security of personal health information and compliance with privacy regulations (such as HIPAA). | *“All client data can be stored in one HIPAA compliant platform”* |
| Trust in technology | | Reference to trust in technology. | *“Some people don't feel comfortable putting medical info online.”* |
| ***Primary Code: Platform Features*** | | | |
| Data Collection | Comprehensive | Reference to versatility of the platform. | *“REDCap can be configured to fit so many needs that it can be invaluable as a tool.”* |
|  | Data administration | Being able to manage administrative tasks with respect to data collection. | *“The ability to integrate multiple inputs of data from a range of sources and being able to maintain administrative research tasks together with the data collection.”* |
|  | Offline Access | Ability to complete surveys offline using mobile apps. | *“Offline data collection when not connected to network.”* |
|  | Familiarity | Study staff’s familiarity with family. | *“It's an established medium in which you can build and record any survey you wish.”* |
| Cost |  | Access to REDcap for free or little to no cost. | *“It's free, and has an open API”* |
| Comparison with Other Platforms | | Comparing REDCap with other commercial tools or paper surveys. | *“it's not a very user's friendly platform compared to 'Survey Monkey' or Google Forms or Qualtrics.”* |
